# Electronic patient-reported outcomes as digital therapeutics for patients with cancer: a narrative review of current practices and future directions

**DOI:** 10.1007/s10147-024-02651-8

**Published:** 2024-11-16

**Authors:** Ken Yamaguchi, Nozomi Higashiyama, Maki Umemiya, Yoshihide Inayama, Ayami Koike, Akihiko Ueda, Rin Mizuno, Mana Taki, Koji Yamanoi, Ryusuke Murakami, Junzo Hamanishi, Masaki Mandai

**Affiliations:** Department of Gynecology and Obstetrics, Graduate School of Medicine and Faculty of Medicine, Kyoto University, 54 Kawahara-cho, Shogoin, Sakyo-ku, Kyoto, 606-8507 Japan

**Keywords:** Digital therapeutics, Electronic patient-reported outcomes, Digital health, Cancer, Quality of life

## Abstract

Improved cancer treatment outcomes have increased the demand for medical care that considers the quality of life of patients with cancer. Patient-reported outcomes (PROs) help assess the quality of life because they involve direct evaluation of the patients. Recently, electronic PROs (ePROs) have been used in clinical cancer care settings in Europe and the United States. Electronic PROs positively affected communication between patients with cancer and healthcare providers, enhanced education, optimized self-management, contributed to healthcare economics, assisted in monitoring adverse events, and improved prognosis. However, challenges such as adherence, burden on healthcare providers, lack of personalized formats, low digital literacy, and implementation costs remain. Therefore, carefully selecting the items to be recorded by ePROs in alignment with specific objectives is essential. Additionally, developing systems using lifelogs—digital records of daily activities—and creating mechanisms that automatically encourage patient behavioral changes based on the reported data are crucial. This review delineates the advantages and challenges of ePROs according to their history and proposes the prospects of ePRO.

## Introduction

With advancements in cancer treatment, improving the prognosis and maintaining and enhancing the quality of life (QOL) of patients with cancer have become essential objectives in cancer care [1]. Patient-centered care, patient-driven support, palliative care, and tumor treatment must be comprehensive throughout the diagnosis, treatment, and post-treatment phases to maintain and improve the QOL of patients with cancer [2–5]. Patient perceptions of adverse events associated with cancer treatment change between therapeutic phases [6]. The physicians assess symptoms using the Common Terminology Criteria for Adverse Events (CTCAE); however, symptoms reported by healthcare providers are reported less frequently and later than those reported directly by patients [7–10]. Therefore, direct symptom evaluation from patients, known as patient-reported outcomes (PROs), is crucial to accurately capture adverse events and QOL. PROs are defined as self-assessment reports of patients and their disease and treatment without external interpretation [11]. Adverse events and QOL deterioration occur even at home, where medical providers are absent. Although physician assessments using the CTCAE correlate with events such as mortality and hospitalization, PROs reflect the daily health status of patients more accurately. Therefore, the complementary use of both methods can provide clinically important information [12].

Recent advancements in digital communication technology have improved accessibility and the accurate capture of patient needs, thereby reducing healthcare disparities by extending medical services beyond clinical settings [13]. Digital health technologies can be used for cancer screening, patient education, shared decision-making, communication, symptom monitoring, and promoting a healthy lifestyle [13]. Therefore, electronic PROs (ePROs)—components of digital health—play a crucial role in enhancing patient outcomes, streamlining healthcare delivery, and supporting patient-centered care. However, challenges such as maintaining adherence, the strain on healthcare providers, the lack of personalized formats, low levels of digital literacy, and the costs associated with implementation still exist. Therefore, this article reviews evidence on ePROs to propose future perspectives.

### History of ePRO clinical studies in oncology

**Fig. 1:**
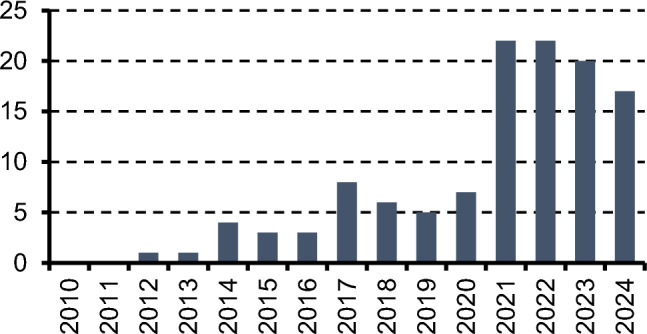
The number of clinical trials describing digital interventions regarding quality of life and cancer in ClinicalTrials.gov. The number of clinical trials describing digital interventions for the quality of life and cancer has increased since 2021

PROs are used in clinical trials for new therapeutic drugs because adverse events and QOL should be evaluated in addition to treatment outcomes in cancer treatment. Between 2007 and 2013, a total of 27% of clinical trials were registered at ClinicalTrials.gov [14]. Review articles on PROs, including ePROs, published between 2013 and 2015 reported improvements in symptom management, supportive care, patient communication, and satisfaction; however, none have reported improvements in QOL [15–17]. Since 2014, the QOL of patients with cancer has reportedly improved owing to ePROs [18–20]. Therefore, attempts have been made to incorporate ePROs, more easily accessible than PROs, into medical care. A literature review of intervention studies assessing digital health from 1999 to 2021 revealed a rapid increase in articles on ePROs after 2013 [21]. An assessment of ClinicalTrials.gov with “Cancer” in the condition or disease field, “QOL” in other terms, and “digital intervention” in intervention or treatment shows that only one clinical trial on the use of ePROs began in 2012, followed by three to four trials per year until 2016, five to eight trials per year from 2017 to 2020, and over 20 trials per year from 2021 (Fig. 1). In a randomized trial involving 100 postoperative patients with lung cancer, symptom monitoring was conducted twice weekly via automated phone calls over 4 weeks. The system automatically alerted healthcare providers when severe symptoms were detected, facilitating timely intervention and symptom control [22]. This system is possible because ePROs are not paper-based. The first report of improvements in overall survival (OS) because of ePROs was published in 2017 [23, 24]. Furthermore, QOL assessments using ePROs have been performed in cutting-edge clinical genomic research [25]. The increased use of PROs in evaluating artificial intelligence (AI) health technologies also demonstrates the importance of patient-centered care, even in the most technologically advanced healthcare systems [26]. This increased use of PROs may be attributed to a greater awareness of their benefits and guidance from regulatory authorities [26].

However, the various methods by which PRO data are analyzed, presented, and interpreted can lead to potential errors and inconsistencies that could adversely affect patient care and outcomes. Therefore, the Setting International Standards in Analyzing Patient-Reported Outcomes and Quality of Life Endpoints in Cancer Clinical Trials-Innovative Medicines Initiative (SISAQOL-IMI) consortium has established recommendations for designing, analyzing, presenting, and interpreting PRO data in cancer clinical trials based on previous SISAQOL activities [27].

### Advantages of ePRO in oncology

#### Communication between patients and healthcare providers

ePRO systems enhance communication between patients and healthcare providers, enabling effective symptom management and timely interventions [28, 29]. A randomized controlled trial (RCT) demonstrated that using ePROs resulted in a 20% increase in patient-provider communication regarding symptoms and side effects [28, 29]. The impact of using the Electronic Self-Report Assessment–Cancer (ESRA-C) in outpatient settings to facilitate discussions between clinicians and patients with cancer regarding symptoms and QOL issues was investigated [30]. The discussions during outpatient visits were more frequent when symptoms and QOL issues were initially reported and clinicians indicated that summaries provided by the ESRA-C were valuable [30]. In the Patient-Reported Outcomes To Enhance Cancer Treatment (PRO-TECT) trial, patients, clinicians, and nurses experienced improved discussions [31].

#### Educating patients and caregivers

ePRO systems provide valuable educational resources, aiding patients and caregivers in understanding and managing their condition, potentially leading to improved health outcomes [32]. ePROs are associated with increased patient empowerment and self-management capabilities, as patients using ePROs require more disease-specific content [32]. A total of 97% of the clinicians noted that ePROs using the PRO-TECT system helped provide the patients with information [31].

#### Patient condition monitoring, self-management, and healthcare costs

ePRO systems facilitate continuous monitoring and support for patient self-management, allowing the early detection of issues and timely interventions. The benefits of ePRO measurement include greater patient preference and acceptability, similar or faster completion times, higher data quality and response rates, and facilitated symptom management [33]. Patient self-management as a component of self-management interventions has been demonstrated to contribute to enhanced self-efficacy. Moreover, increased patient self-efficacy has been associated with enhancements in health-related QOL [34].

eRAPID, which was developed in the United Kingdom, is an online eHealth system for patients with cancer to self-manage adverse events during and after treatment [35]. A phase-III trial evaluated the impact of eRAPID on symptom control, healthcare utilization, patient self-efficacy, and QOL in patients receiving chemotherapy for colorectal, breast, or gynecological cancers. Patients using eRAPID reported higher self-efficacy and better health status scores on the EQ5D-VAS at 18 weeks than patients who did not use eRAPID. Patient adherence correlated with clinical data usage and was associated with improved QOL at 12 weeks [36].

Furthermore, ePRO implementation can reduce costs by decreasing emergency visits and hospital admissions through improved symptom management [4, 37, 38].

#### Managing adverse events and QOL

The regular use of ePRO systems is associated with better management of adverse events and increased patient QOL during treatment [39, 40]. The ESRA-C intervention group demonstrated significantly improved symptom relief compared to the control group [18]. Another randomized study compared a control group that solely used a fully automated ESRA-C with an intervention group that received results delivered to their homes or hospitals. The intervention group showed an estimated 1.53-point reduction in Symptom Distress Scale-15 (SDS-15) scores compared to the control group [41]. A multicenter RCT using Electronic Monitoring of Symptoms and Syndromes Associated with Cancer (E-MOSAIC) showed that overall QOL—which was assessed by using items from the EORTC-QLQ-C30—showed favorable trends in the intervention group, with significant improvement in symptom management. Cancérologie Parcours Région Ile de France-Remote Patient Monitoring Systems (CAPRI-PRMS) is a nurse navigator-led program developed in France [42]. A phase-III trial randomly assigned patients receiving approved oral anticancer agents or molecular targeted therapies to either an intervention group, which received nurse navigator-led follow-up through a combination of a web portal and smartphone application in addition to standard care for 6 months, or a control group, which underwent regular symptom monitoring by treatment providers. The intervention did not significantly alter the EORTC-QLQ-C30 Global Health Score. However, it significantly improved the Patient Assessment of Chronic Illness Care score (2.94 vs. 2.67), reduced hospitalization days (2.82 vs. 4.44 days) and decreased treatment-related Grade 3 or higher toxicities (27.6 vs. 36.9%) [43]. eRAPID showed that the intervention group had improved FACT-PHYSICAL WELLBEING at 6 and 12 weeks compared with the control group. Symptom Tracking and Reporting (STAR) e-mail nurses and patients alerts for severe symptoms or deterioration. Compared to the baseline, the intervention group showed a significantly improved QOL compared to the control group (7.1 points vs. 1.4 points in EQ-5D-5L), lower emergency room visit rates (45 vs. 49%), and prolonged chemotherapy duration (8.2 vs. 6.3 months). In the PRO-TECT trial, a multicenter cluster randomized trial, the EORTC-QLQ-C30 scores in the intervention group compared to the control group at 3 months showed significant improvements with approximately 2.5 times higher QOL [44]. Moreover, the probability of experiencing clinically meaningful benefits favored the PRO group regarding physical functioning, symptom control, and health-related QOL (HRQOL; odds ratios 1.35, 1.50, and 1.41, respectively) compared to the standard care group [44]. In an intention-to-treat analysis assessing CANKADO PRO-React, the cumulative incidence of QOL deterioration was approximately 1.4 times lower in the CANKADO treatment group than in the control group [45]. Mika is a digital therapy aimed at alleviating pain associated with cancer and its treatments, to improve the QOL of patients [46]. Compared with the control group, the intervention group showed significant reductions in pain, depression, anxiety, and fatigue [46].

A meta-analysis conducted from 2016 to 2021 involving 2058 adult patients showed that remote patient monitoring software improved QOL and reduced physical symptom burden during cancer treatment [13]. Furthermore, a meta-analysis of 32 RCTs involving 7888 patients demonstrated that digital self-management symptom interventions significantly reduced symptom burden (effect size [ES] = − 0.230), pain (ES = − 0.292), fatigue (ES = − 0.417), anxiety (ES = − 0.320), and depression (ES = − 0.261) [13].

These studies highlight the significant potential of ePROs in enhancing symptom management, improving QOL, and reducing the burden of physical and psychological symptoms in patients with cancer.

#### Therapeutic outcomes

Digital therapeutics (DTx) and ePROs increase treatment adherence and enhance medical safety and quality, thereby improving treatment outcomes [4, 38, 40, 47]. The physician-reported symptoms such as fatigue, nausea, and constipation have been significantly associated with mortality and emergency room visits compared with those reported through ePROs [7], suggesting that physician-interpreted symptom reporting may better capture the more severe conditions of patients. ePRO-driven DTx can reduce symptom burden, unplanned hospitalizations, and medication non-adherence and improve adverse event management, QOL, and survival rates [4, 38]. The CAPRI-PRMS trial indicated that the relative intensity of treatment dosing was higher in the intervention group than in the control group (93.4 vs. 89.4%) [42].

Using ePROs to improve OS was first reported in 2017 [23, 24]. The STAR clinical trial is a randomized comparative trial with QOL as the primary outcome, and retrospective prognostic results have been reported [20]. The STAR group observed a significant improvement in median OS by approximately 5 months (median OS: intervention group 31.2 months vs. usual care group 26 months) [23]. Based on these findings, the PRO-TECT trial was a large-scale randomized trial conducted across multiple centers [44]. An electronic follow-up application (e-FAP) has proven highly reliable, detecting recurrences an average of 5 weeks earlier than regular imaging diagnostics [48, 49]. Subsequently, an RCT validating its prognosis revealed a median OS of 19 months (95% confidence interval [CI] 12.5 to not calculable) in the e-FAP group compared to 12 months (95% CI 8.6–16.4) in the control group (hazard ratio [HR] = 0.32, 95% CI 0.15–0.67) [24]. Subsequent 2-year follow-up reports added a median OS of 22.5 months in the e-FAP group vs. 14.9 months in the control group (without crossover termination; HR = 0.59 [95% CI 0.37–0.96]). Upon terminating crossover, the median OS was 22.5 months in the e-FAP group vs. 13.5 months in the control group (HR = 0.50 [95% CI 0.31–0.81]) [50].

### Challenges of ePROs

#### Adherence

Adherence is a barrier to using ePROs. The barriers to ePRO use include patients, clinicians, and health system levels. Thirty percent of patients consistently failed to use ePRO systems because of adherence barriers. Additionally, adherence rates varied substantially based on demographic factors, with lower adherence observed among older adults and those with a lower socioeconomic status. Patient-level barriers include education, digital literacy, language barriers, and lack of understanding of the necessity of ePRO use. Physician-level barriers include being busy, difficulty making final decisions based on ePRO results, and uncertainty about integrating ePRO results into clinical practice. System-level barriers include a lack of technical support within hospitals and economic constraints [51].

#### Overburden on healthcare providers

Using ePRO systems can increase the workload of healthcare providers, who must review and act on the data, potentially leading to burnout and decreased efficiency [52, 53]. Although ePROs can facilitate comprehensive healthcare by assessing QOL and directly treating the primary condition, the healthcare provider burden remains an issue. The PRO-TECT trial highlighted challenges in the workload of nurses receiving alerts, prompting the development of algorithms to identify alerts with a high likelihood of non-urgency [52]. Ninety-one percent of the patients responded to weekly surveys, with alerts generated in 34% of the surveys and immediate nursing actions were taken in 59% of the alerts. Patients perceived 10% of the alerts as urgent. Nurses often classified alerts as urgent when patients reported worse symptoms than the previous week. The developed algorithm identified 38% of alerts as likely nonurgent [52].

#### Lack of formats in recent technologies

Numerous PROs exist, including those that report QOL and adverse events. The Patient-Reported Outcomes version of the Common Terminology Criteria for Adverse Events (PRO-CTCAE^®^), developed by the National Cancer Institute [54], is frequently used to report adverse events. Recording PROs on days when deterioration occurs can quickly convey the symptoms to healthcare providers because the side effects of cancer treatment can fluctuate daily. However, the recommended recall period for the PRO-CTCAE^®^ is 1 week [55], limiting the real-time monitoring of adverse events in clinical practice. PRO tools such as the EORTC-QLQ and FACT are commonly used to evaluate the QOL of patients with cancer. However, these tools are copyrighted and not available for commercial use. Although paper-based PROs are relatively easy to collect, patients must answer numerous questions, making frequent data collection, and remote monitoring challenging.

#### Lack of technical support in hospitals

Many healthcare systems lack the necessary technical infrastructure and support to effectively implement and maintain ePRO systems [51]. Forty percent of healthcare facilities reported insufficient technical support as a significant barrier to the successful deployment of ePRO systems [51], hindering the integration of ePROs into routine clinical practice.

#### Economic concerns

The costs associated with implementing and maintaining ePRO systems can be a substantial barrier, particularly in resource-limited settings where financial constraints are a primary concern [51]. The initial setup and ongoing maintenance costs of ePRO systems were excessive, posing a barrier to widespread adoption [51]. Additionally, economic constraints are key challenges in sustaining these systems in low-income healthcare settings [51].

### Future prospects and proposal of new technologies and approaches

#### Symptoms that ePRO should monitor

**Fig. 2:**
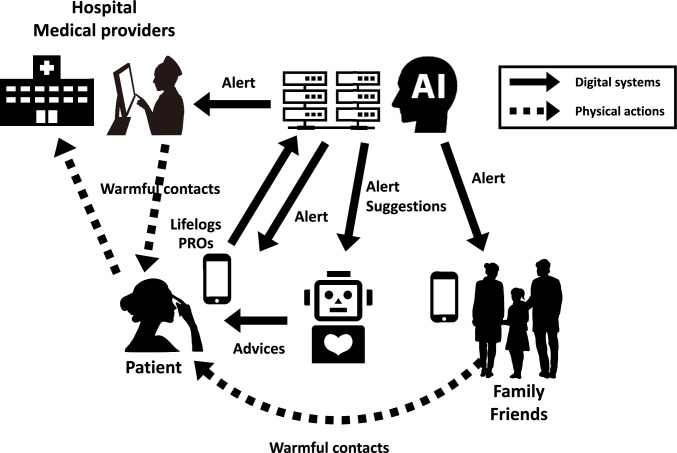
Patient care using new technologies and approaches. The number of clinical trials describing digital interventions for the quality of life and cancer has increased since 2021

Early ePROs often used validated QOL scales. The EORTC-QLQ-C30 and Hospital Anxiety and Depression Scale used touchscreen technology in a 2004 intervention trial [56]. The ESRA-C also uses validated measures such as the SDS-15 and EORTC-QLQ-C30 [18]. However, minimizing the number of recorded symptoms is crucial for adherence (Fig. 2). Table 1 summarizes RCTs using the original symptom records via ePRO. Some ePROs allow for selecting and recording 11–17 symptoms from a broader list. Although specific symptoms vary according to disease and study objective, dyspnea and fatigue were consistently incorporated into four or more ePROs among 10 ePROs, followed by appetite loss, diarrhea, nausea, depression, pain, constipation, fever, vomiting, insomnia, anxiety, and neuropathy (Table 2). These symptoms are presumed to affect the QOL of patients across various cancer types.

**Table 1:** List of randomized clinical trials using electronic patient-reported outcomes that record selected symptoms

ePRO or ClinicalTrials.gov	Patient population	Trial design	Sample size	Study arms	Number of recorded symptoms	Primary outcome	Summarized findings	Publication, trial setting
E-MOSAIC	Patients receiving anticancer treatment in palliative intention	RCT	102	E-MOSAIC intervention with the longitudinal monitoring sheet given to physicians vs. standard care	9–12	QOL	Favor of the intervention for QOL, symptoms, communication, and coping	Strasser et al. [19], Switzerland
NCT02004496	Patients receiving chemotherapy for breast cancer	RCT	127	Mobile or web-based app with physician supervision vs. mobile or web-based app without physician supervision vs. standard care	Selection from 48	QOL	Declined functional status was reported in the control and unsupervised app-using groups; however, no significant decline was observed in the supervised app-using group More symptoms were reported in the app-using group than in the control group. More symptoms were reported in the unsupervised group than in the supervised group Higher satisfaction with care was reported in the supervised app-using group than in the other groups	Egbring et al. [9], Switzerland
STAR	Patients receiving chemotherapy for advanced solid cancer and metastatic disease	RCT	766	Weekly patient-adapted CTCAE assessments vs. usual care	12	QOL	Improved HRQOL was reported among patients who completed the patient-adapted CTCAE assessments Significantly lower emergency room admission for patients in the digital therapeutics group than in the usual care group Longer chemotherapy periods in the digital therapeutics group than in the usual care group Improved OS	Basch et al. JCO 2016 Basch et al. JAMA 2017, US
e-FAP Moovcare	Patients with advanced-stage lung cancer undergoing systemic therapy	RCT	121	Weekly electronic follow-up application (e-FAP) vs. usual care	Weight and 11 symptoms	OS	Improved OS and higher performance status were observed at relapse in the intervention group than in the usual care group	Denis et al. JNCI [24] Denis F et al. JAMA [50], France
Oncokompas	Post-treatment patients with head and neck cancer, colorectal cancer, breast cancer, Hodgkin’s lymphoma, or non-Hodgkin’s lymphoma after treatment with curative intent (all treatment modalities)	RCT	625	“Oncokompas” app vs. standard care	18 physical, four psychological, eight social, six lifestyle, one existential questions, and tumor-specific modules (6–11)	Patient activation (knowledge, skills, and confidence for self-management	Better QOL was observed in the app-using group than in the control group at 6 months of follow-up Less pain in the mouth, coughing, swallowing difficulty, and more social eating were observed in head and neck cancer survivors in the app-using group Less weight change was observed in colorectal cancer survivors in the app-using group than in the control group Less emotional impact was observed in lymphoma survivors in the app-using group than in the control group No difference in reported symptoms was observed in breast cancer survivors No significant difference was observed in the intervention and control groups regarding health management knowledge, attitude, self-efficacy, and supportive care needs More app usage was reported in patients with higher education, having a partner, and currently working	Hout et al., 2019, Hout et al. Acta Oncol 2021, Netherlands
Interaktor	Patients scheduled to undergo pancreaticoduodenectomy due to a suspected malignancy	RCT	59	“Interaktor” app vs. standard care	14	QOL, self-care activity	Higher emotional function, less nausea, pain, appetite loss, constipation, pancreatic pain, flatulence, and weight loss. Worries and fewer hepatic symptoms were observed in the app-using group than in the control group at six weeks post-operation No significant difference was observed between the app-using group and the control on engagement in self-care activities	Gustavell et al., 2019, Sweden
ChemOtheRapy Assistant (CORA)	Patients with diverse malignancies who were prescribed oral therapy for cancer	RCT	181	CORA app vs. standard care	17	Adherence, symptom burden, QOL	No significant difference was observed in all study measurements Higher improvement in adherence rate in app-using participants with higher anxiety and poor baseline medication adherence were observed	Greer et al., 2020, US
BPSS	Patients with breast cancer who had undergone anthracycline- or taxane-based chemotherapy regimens	RCT	95	“BPSS” web app vs. standard care	12	QOL (psychological distress)	No significant difference between app-using and control groups was observed on depression subscales or health literacy skills Less anxiety was observed in the app-using group than in the control group The medical staff underestimated some grade 3 symptoms compared to the patient’s records	Handa et al. [10], Japan
SOFIA	Patients who received immune checkpoint inhibition therapy	RCT	71	Intervention group (SOFIA) vs. usual care	Eleven physical and up to nine mental ePROs	Feasibility of SOFIA	Higher feasibility and acceptance in the SOFIA group than in the control group Better HRQOL and role functioning and less depression, distress, and appetite loss were observed in the SOFIA group post-intervention No significant differences were observed regarding medical data, the utilization of supportive care services, or survival	Sauer et al. Cancer 2020, Germany
Msymptom	Patients with breast cancer receiving adjuvant and neoadjuvant chemotherapy treatment for the first time	RCT	57	“Msymptom” app vs. standard care		Symptom control, QOL	Lower physical symptom burden was observed in the app-using group than in the control group Higher scores were observed on the EORTC QOL-C30 symptom scale and nausea/vomiting score in the app-using group Higher scores on EORTC QOL-BR23 sexual function and sexual pleasure subscales were observed in the app-using group	Öztürk et al., 2021, Turkey
eRAPID	Patients with colorectal, breast, or gynecological cancer undergoing chemotherapy	RCT	508	Weekly eRAPID vs. usual care	17	Symptom control	Improved physical well-being and self-efficacy were observed in the eRAPID group Higher confidence in health management and self-rated overall health were observed in the app-using group	Absolom et al. JCO [36], UK
PRO-TECT	Patients with metastatic cancer undergoing systemic therapy	Cluster RCT	1191	Weekly PRO-CTCAE assessments vs. usual care	11	OS	Improved physical function, symptom control, and HRQOL (OS pending) by weekly PROCTCAE assessments	Basch et al. JAMA [44], US
CANKADO PRO-React	Patients with HR^+^/HER2^−^ locally advanced or metastatic breast cancer	RCT	499	CANKADO-active arm vs. CONKADO-inactive inform arm	13	QOL (psychological distress)	Favorable QOL in CANKADO-active arm	Harbeck et al. Ann Oncol [45], Germany
Mika	Adult patients with malignant tumors	RCT	218	Mika-App and usual care vs. usual care	Selection from 58 options	QOL	Higher reductions were observed in distress, depression, anxiety, and fatigue were observed in the intervention group than in the control group	Spring et al. J Med Internet Res [46], Germany

*CTCAE* Common Terminology Criteria for Adverse Events, *e-FAP* electronic follow-up application, *HRQOL* health-related quality of life, *QOL* quality of life, *OS* overall survival, *RCT* randomized controlled trial

**Table 2:** Selected symptoms that were applied in the electronic patient-reported outcomes

	Total	E-MOSAIC	STAR	e-FAP	Oncokompas	Interaktor	BPSS	SOFIA	eRAPID	PRO-TECT	CANKADO
Dyspnea/shortness of breath	9	●	●	●	●	●		●	●	●	●
Fatigue	9	●	●	●	●	●	●	●	●		●
Appetite loss	8	●	●	●	●		●		●	●	●
Diarrhea	8		●		●	●	●	●	●	●	●
Nausea	8	●	●		●	●	●		●	●	●
Depression	6	●		●	●	●		●		●	
Pain	6	●	●	●	●	●			●		
Constipation	6		●		●	●	●		●	●	
Fever/chills	6			●		●	●	●	●		●
Vomiting	6		●			●	●		●	●	●
Insomnia/drowsiness	5	●			●	●			●	●	
Anxiety	4	●			●	●		●			
Neuropathy	4		●			●	●		●		
Cough/dry cough	3		●	●				●			
Mucositis/oral problems	3					●	●		●		
Dysuria/reduced urinary output	2		●					●			
Hot flashes/night sweats	2		●								●
Joint pain/muscle pain	2						●	●			
Rash/skin toxicity	2							●	●		
Skin changes	2						●				●
Visual disturbances	2				●						●
An occurrence of a lump under the skin	1			●							
Appearance or increase of blood in sputum	1			●							
Bleeding	1										●
Falls	1									●	
Financial challenges	1									●	
Flu-like symptoms	1								●		
Hair loss	1										●
Headache	1						●				
Melaena	1							●			
Nose bleeds	1								●		
Overall well-being	1	●									
Palmar-plantar erythema	1								●		
Performance status	1									●	
Psychological distress	1							●			
Stomatitis	1										●
Sudden face swelling	1			●							
Swelling/pain/redness in the arm	1					●					
Thrombocytic purpura	1								●		
Voice changing	1			●							
Weakness	1							●			
Weight	1			●							
Yellow coloring	1							●			
Others	3	●			●			●			

*e-FAP* electronic follow-up application, *E-MOSAIC* Electronic Monitoring of Symptoms and Syndromes Associated with Cancer, *PRO-TECT* Patient-Reported Outcomes To Enhance Cancer Treatment, *STAR* Symptom Tracking and Reporting

#### Use of lifelogs

Methods unrelated to patient adherence should be used and digital information related to patients’ daily lives (lifelogs) helps evaluate QOL (Fig. 2). Using lifelogs can minimize the influence of adherence and language barriers. Integrating real-world big data in a reliable cancer research environment may assist clinicians and researchers in developing new models for predictive and preventive algorithms and personalized care [57]. Diverse lifelogs were collected from approximately 100 patients with gynecological cancer, and associations with QOL-related lifelogs were observed, particularly with heart rate variability (unpublished). Heart rate variability, measured from R waves and their fluctuations, can be assessed using a smartphone camera. Thus, leveraging digital information from wearable devices and mobile applications enables cancer healthcare management without the patient recording the data, thereby representing a future direction of cancer treatment. Illustrating how real-world big data can contribute to the development of new models for predictive and preventive algorithms and personalized care is crucial.

#### Automatic alert systems

In 2022, the European Society for Medical Oncology released clinical practice guidelines for patient-reported outcome measurements [58]. These guidelines specifically address the recommendations of ePRO for patients with cancer. The automatic alert functions that notify clinicians of severe or worsening symptoms are recommended for patients undergoing active cancer treatment [58]. However, overburdening healthcare providers with automatic alert functions is challenging for clinicians. Therefore, an automatic alert system for patients can be developed to reduce the burden on healthcare providers (Fig. 2).

CANKADO PRO-React represents an advanced next-generation ePRO, suggesting actions to patients automatically rather than alerting healthcare providers. These recommendations encourage behavioral changes in patients categorized into observation until the next visit (Level 1), prompt contact with the center (Level 2), or immediate contact or emergency visit (Level 3) [45]. Mika is a next-generation ePRO comprising three modules: Check-up, Discover, and Journeys [46]. The Check-up module monitors pain and symptoms, enabling electronic outcomes reported by patients to be shared and discussed with healthcare providers. The Discover module provides coaching through articles and videos on cancer types, treatments, psychological health, physical activity, diet, and socioeconomic issues. In the Journeys module, AI algorithms in the Mika application customize content for each patient based on cancer type, treatment stage, and the reported symptom nature and severity, prompting behavior change and ensuring personalized support for each individual [46]. Oncokompas, a Dutch-approved ePRO, is divided into three components: Measure, Learner, and Act components [59]. However, no significant differences were observed in patient activation (knowledge, skills, or confidence in self-management) between the intervention and control groups [59]. Nevertheless, it suggested effectiveness for HRQOL in cancer survivors with moderate self-efficacy, high personal control, and high health literacy scores, particularly for specific symptoms such as mouth pain in head and neck cancer, social eating, swallowing, coughing, and weight in colorectal cancer [60].

ePROs have been developed to promote behavioral changes in patients and reduce the burden on healthcare providers. However, patients expressed a desire for clinicians to acknowledge the results of an ePRO system called MyChristie-MyHealth [61], indicating the crucial role of physicians in patient care. ePRO-promoting behavioral change in patients has demonstrated improvement in symptoms and QOL enhancement. However, safety evaluations and patient satisfaction are crucial and future research should verify their impact on treatment outcomes.

## Conclusions

ePROs enhance patient–provider communication, educate patients and caregivers, facilitate the monitoring of patient conditions and self-management, and improve adverse event management and QOL, thereby potentially improving treatment outcomes and reducing healthcare expenditures. To fully capitalize on the advantages of ePROs, future advancements should prioritize developing lifelogs independent of adherence to assess patient symptoms and QOL and create automatic alert systems for patients to alleviate the burden on healthcare providers while rigorously evaluating their safety and efficacy.
